# Editorial for “Asthma and Its Impact in Adolescents” Special Issue

**DOI:** 10.3390/children10111811

**Published:** 2023-11-15

**Authors:** Roberto W. Dal Negro

**Affiliations:** National Centre for Respiratory Pharmacoeconomics and Pharmacoepidemiology, Research & Clinical Governance, 37124 Verona, Italy; robertodalnegro@gmail.com

Bronchial asthma is a chronic disease related to the atopic condition in most cases but also to other factors (such as infectious diseases; social, economic, environmental, and occupational conditions; exposure to biological irritants; and/or chemical aggressions).

The incidence of asthma has progressively increased during the last few decades, also in young individuals, though characterized by variable evolution. Regardless of the widespread diffusion of international guidelines, the morbidity of bronchial asthma is still causing relevant limitations in their quality of life and represents a socio-economic burden for their families.

The present Special Issue, “Asthma and its Impact in Adolescents”, is dedicated to specific topics to broaden the current approach to asthma in young patients and favor their clinical control and self-management.

Heterogeneous conditions affecting children and adolescents suffering from persistent asthma have been widely reviewed, together with the role of comorbidities and other associated factors involved in their preschool age. Particular attention has been dedicated to the increasing preference for vaping in adolescents and to the related risk of uncontrolled asthma.

The peculiar role of the hydration status of asthmatic teenagers (usually due to insufficient daily water intake) has been pivotally described and compared to the behavior of normal controls of comparable age. Dehydration proved able to work systematically as a critical risk factor of asthma instability but was unfortunately underestimated and unknown by families and caregivers. On the other hand, the use of cold, dry air inducing similar mucosal stress actually represents a provocation test for investigating asthma in pediatric subjects. Also, the risk factors for lung function decline in pediatric asthma and the relationship between lung function and quality of life in young asthmatics were reviewed.

The problem of the low adherence of asthmatic adolescents to regular pharmacological anti-asthma strategies, and to inhalation treatments in particular, has been confronted. This dangerous attitude is frequently underestimated in clinical practice and too frequently neglected by young patients, their families, and sometimes also by their caregivers. As the knowledge of the main factors affecting the effectiveness of inhalation therapies is still generally quite low, part of the present Special Issue is dedicated to these peculiar aspects. In particular, the effects of patients’ adherence on long-term outcomes have been described in asthmatic adolescents from the clinical and the economic point of view, together with the probability of lung function to predict the most effective inhaler (Dry Powder Inhalers in particular) to choose for the patient’s daily use, according to a personalized approach.

The results of novel technologies aiming to support and favor asthma education and self-management in teenagers have been reported and commented on. Moreover, results of the increasing strategy based on biological therapies introduced in childhood asthma for containing airway remodeling have also been discussed.

Finally, to address the crucial question, “Are adolescents with asthma ready for transition of care?”: at present, parental involvement and communication with providers seem inadequate in children and adolescents with asthma, while parents and providers are insufficiently engaged and prepared for the transition of care and for ensuring a proper and long-term continuity of asthma care.

The main emerging message is that further effort is still needed to optimize the approach to the asthma of adolescents.

In the hope that the content of the present Special Issue may contribute to improving the comprehension, awareness, and proper home management of adolescent asthma, I would like to thank all the authors for their valuable scientific contributions.

